# Erosion of natural darkness in the geographic ranges of cacti

**DOI:** 10.1038/s41598-018-22725-8

**Published:** 2018-03-12

**Authors:** Maria Eugenia Correa-Cano, Bárbara Goettsch, James P. Duffy, Jonathan Bennie, Richard Inger, Kevin J. Gaston

**Affiliations:** 1 Environment and Sustainability Institute, University of Exeter, Penryn, Cornwall, TR10 9FE UK; 2 International Union for Conservation of Nature, Global Species Programme, The David Attenborough Building, Pembroke Street, Cambridge, CB2 3QZ UK

## Abstract

Naturally dark nighttime environments are being widely eroded by the introduction of artificial light at night (ALAN). The biological impacts vary with the intensity and spectrum of ALAN, but have been documented from molecules to ecosystems. How globally severe these impacts are likely to be depends in large part on the relationship between the spatio-temporal distribution of ALAN and that of the geographic ranges of species. Here, we determine this relationship for the Cactaceae family. Using maps of the geographic ranges of cacti and nighttime stable light composite images for the period 1992 to 2012, we found that a high percentage of cactus species were experiencing ALAN within their ranges in 1992, and that this percentage had increased by 2012. For almost all cactus species (89.7%) the percentage of their geographic range that was lit increased from 1992–1996 to 2008–2012, often markedly. There was a significant negative relationship between the species richness of an area, and that of threatened species, and the level of ALAN. Cacti could be particularly sensitive to this widespread and ongoing intrusion of ALAN into their geographic ranges, especially when considering the potential for additive and synergistic interactions with the impacts of other anthropogenic pressures.

## Introduction

Concern is being widely expressed as to the negative environmental implications of the introduction of artificial light at night (ALAN), through the use of electric lighting (including, but not limited to, street lighting^1–5^). The reasons are twofold. First, ALAN has rapidly become extremely widespread^5,6^, continues to spread at a fast rate^2^, and is increasingly taking more problematic forms (e.g. the progressive shift from narrow to broad spectrum lighting^7^). Second, empirical studies have demonstrated biological impacts of ALAN from the molecular to the ecosystem level^8^. Effects have been identified on the physiology^9^, behavior^10,11^, reproductive success^10^ and mortality^12^ of a wide range of species, on their abundance and distribution^13^, and in turn on community structures^14^. Although substantially less attention has been paid to the impacts of ALAN on plants than on animals, direct effects of artificial nighttime lighting have been demonstrated in horticultural research. For example, Park *et al*.^15^ showed that morphogenesis and flowering of individuals of *Dendranthema grandiflorum* were significantly altered by interrupting the night with different light spectra during the last 2 hr of the normal dark period. Likewise, Kim *et al*.^16^ showed that night interruption for four months in some varieties of the orchid *Cymbidium* sp. triggered flowering and increased plant size within 2 years, while individuals under natural darkness conditions did not flower during that time (for other examples of impacts on plant productivity and phenology see^17–19^). In addition, indirect effects on plants can plainly occur through impacts of ALAN on animals and their patterns of herbivory, pollination and seed dispersal^20,21^.

What has largely been missing from discussion to date of the impacts of ALAN has been an understanding of the proportion, and location, of species that are likely to be affected^5^. The first step here is to determine the relationship between the occurrence of, and trends in, ALAN and that of the geographic ranges of species in particular taxonomic groups. Key questions include how many species are experiencing ALAN somewhere in their geographic range, how extensive this influence is, how it changes with the size of the geographic range, how the distribution of ALAN interacts with that of species richness, and how all of these patterns are changing with changes in the levels of ALAN. To date, the only attempt to address these issues has been for terrestrial mammals, which revealed that most species are experiencing ALAN in some part of their geographic range, that in the majority of cases ALAN is increasing, and that ALAN may contribute to the patterns of risk of extinction of species^22,23^. Studies of many other taxa are plainly required however before any general conclusions can be drawn. Amongst plants this is challenging given the paucity of taxonomic groups for which global geographic ranges have been mapped for all or most of the species.

**Figure 1:**
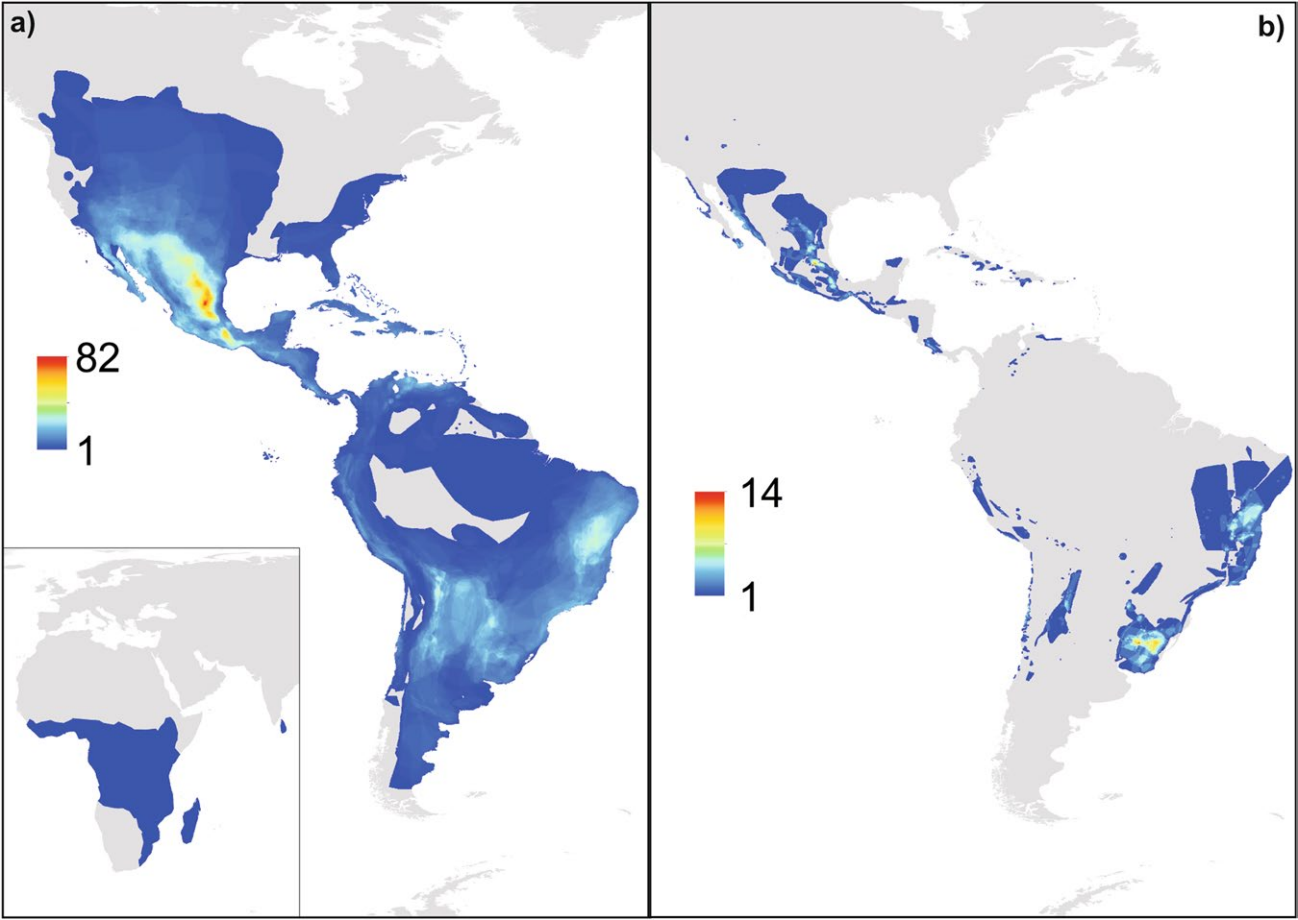
Cactus species distribution. Richness maps generated using cactus distribution maps from the Global Cactus Assessment^25^ in R^63^ using the ‘raster’ package^64^. Final layout made in ArcGis^69^. Maps are presented under a Behrmann equal-area projection. (**a**) cactus species richness distribution. Note that *Rhipsalis baccifera* is the only species occurring in Africa and Sri Lanka, however, the occurrence of a single species in the Americas does not correspond only to *Rhipsalis baccifera*, but to areas where only one cactus species occurs (**b**) threatened cactus species richness distribution.

Here, we address the above questions about the relationship between the occurrence of, and trends in, ALAN and that of the geographic ranges of species, using the morphologically heterogeneous family Cactaceae (the cacti) as a case study. Unusually for a diverse (c.1500 species^24^) plant taxon, the global geographic ranges for the vast majority of extant species of cacti have recently been mapped as part of an assessment of their conservation status, and their threat status and use by people have also been determined^25^. The group is of particular interest for several reasons. First, naturally distributed almost entirely on the American continent (with the exception of *Rhipsalis baccifera* which is the only species naturally distributed in Africa and Sri Lanka) and occurring across a wide range of climatic and ecological conditions^26^, it is somewhat emblematic of, and predominantly distributed in, arid lands (Fig. 1a). Globally, these ecosystems have been shown to be disproportionately influenced by ALAN^27^. Second, the group has significant socioeconomic and cultural importance, with 57% of all known cactus species being utilized by people^25^, and managed in wild and in anthropogenic created spaces (e.g. agricultural lands, pasture, and backyards^28–30^). Third, cacti are amongst the most threatened of any species-rich taxonomic group (animal or plant) to have been formally assessed to date, with the predominant documented threat processes being land conversion to agriculture and aquaculture, harvesting from the wild and, notably in the present context, residential and commercial development^25^ (see Fig. 1b for the distribution of threatened cactus species richness). Finally, there is evidence to suggest that ALAN might have an array of both direct and indirect effects on cacti (see Discussion). Direct effects include influences on germination and on time of seed quiescence^31^. Indirect effects of particular concern are those of ALAN on pollinators and dispersers (e.g. bats and insects^32^).

## Results

**Figure 2:**
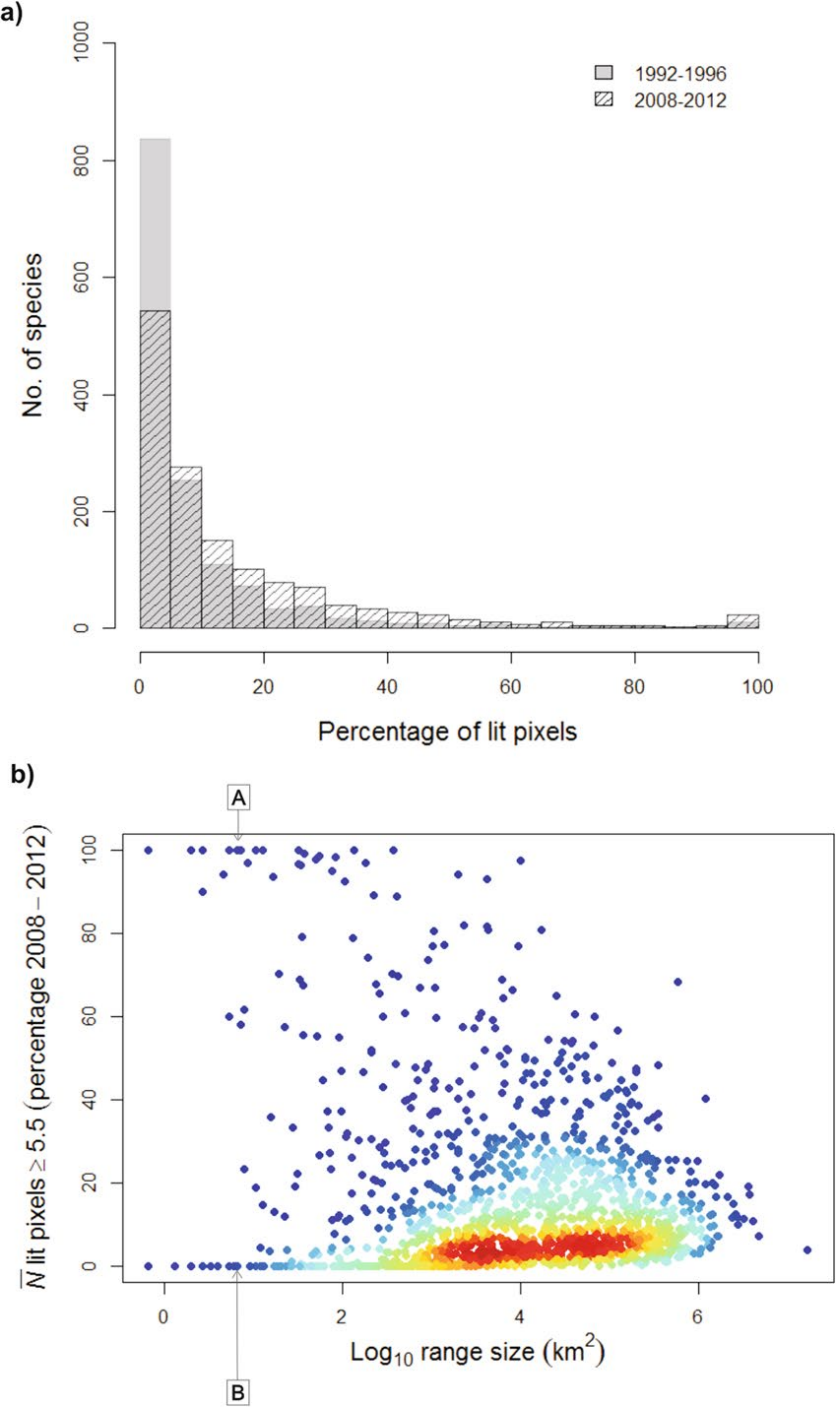
Percentages of the ranges of cactus species lit at night. (**a**) Frequency distribution of the number of cactus species with different percentages of their geographic range lit for the periods 1992–1996 and 2008–2012; (**b**) The relationship between the average percentage of the range of each cactus species ($\bar{N}$) that was lit in 2008–2012 and its range size. Species with similar small range sizes (A) *Cleistocactus pycnacanthus* and (B) *Turbinicarpus alonsoi*. Red indicates the highest density of species in the area of the plot (each dot equals one species). Blue the lowest density. The scatterplot with heat-density colors was created using the ‘LSD’^66^, ‘MASS’^67^, and ‘colorRamps’^68^ packages in R^63^.

Of the 1,435 species analysed, a high percentage (80.7%) had some areas of detectable ALAN within the bounds of their geographic ranges in 1992. This increased to 89.7% of species in 2012 (Table 1). In both years we found species with their geographic ranges lit throughout, i.e. every pixel within their range had digital number (DN) values ≥5.5 (0.6% of species in 1992 and 1.6% of species in 2012, Table 1). The spread of ALAN in these two time periods can indirectly be seen through the species with no lit pixels in their ranges, as in 1992 species with large distribution areas were under the threshold of darkness but in 2012 large ranges no longer appeared as entirely dark (Table 1). The overall trend of increasing erosion of natural darkness across the geographic ranges of cacti was apparent when comparing the percentages of their geographic ranges that were lit in different periods (Fig. 2a). During 1992–1996 more than 800 species were experiencing ALAN across, on average, less than 5% of their range. By 2008–2012 the number of species experiencing the same percentage of light across their ranges declined to nearly 600 species (Fig. 2a). Unsurprisingly, those species with geographic ranges with detectable levels of ALAN in all pixels had small geographic ranges (from 1–25 pixels), although some species with small geographic ranges had no detectable levels of ALAN (Fig. 2b). For example, the range of *Cleistocactus pycnacanthus* (16 pixels) was lit throughout, whilst that of *Turbinicarpus alonsoi* was not lit at all (Fig. 2b). Overall, there was a triangular relationship between the percentage of the range of a species that was lit and its range size, with some species across the breadth of range sizes being largely untouched by ALAN, and the maximal percentage of the range that was lit declining as ranges increased in size (Fig. 2b). It is noticeable that the higher density of species of medium to large range sizes (represented by the red colour in Fig. 2b) were those with on average less than 20% of lit pixels, although species with such range sizes could have up to 100% of lit pixels.

**Table 1:** The number of cactus species with lit pixels (DN ≥5.5) in their geographic range, with no-detectable light in their range and only lit pixels in their range in 1992 and 2012, and the variation in the range sizes of the species in these different groups. Total number of cactus species analysed = 1,435.

	Number of species		Range size (No. of pixels)	
	1992	2012	1992	2012
With lit pixels in their range	1,158	1,287	3–25,969,250	1–25,969,250
With no lit pixels in their range	278	149	1–118,348	1–16,681
With only lit pixels in their range	8	23	3–202	1–567

**Figure 3:**
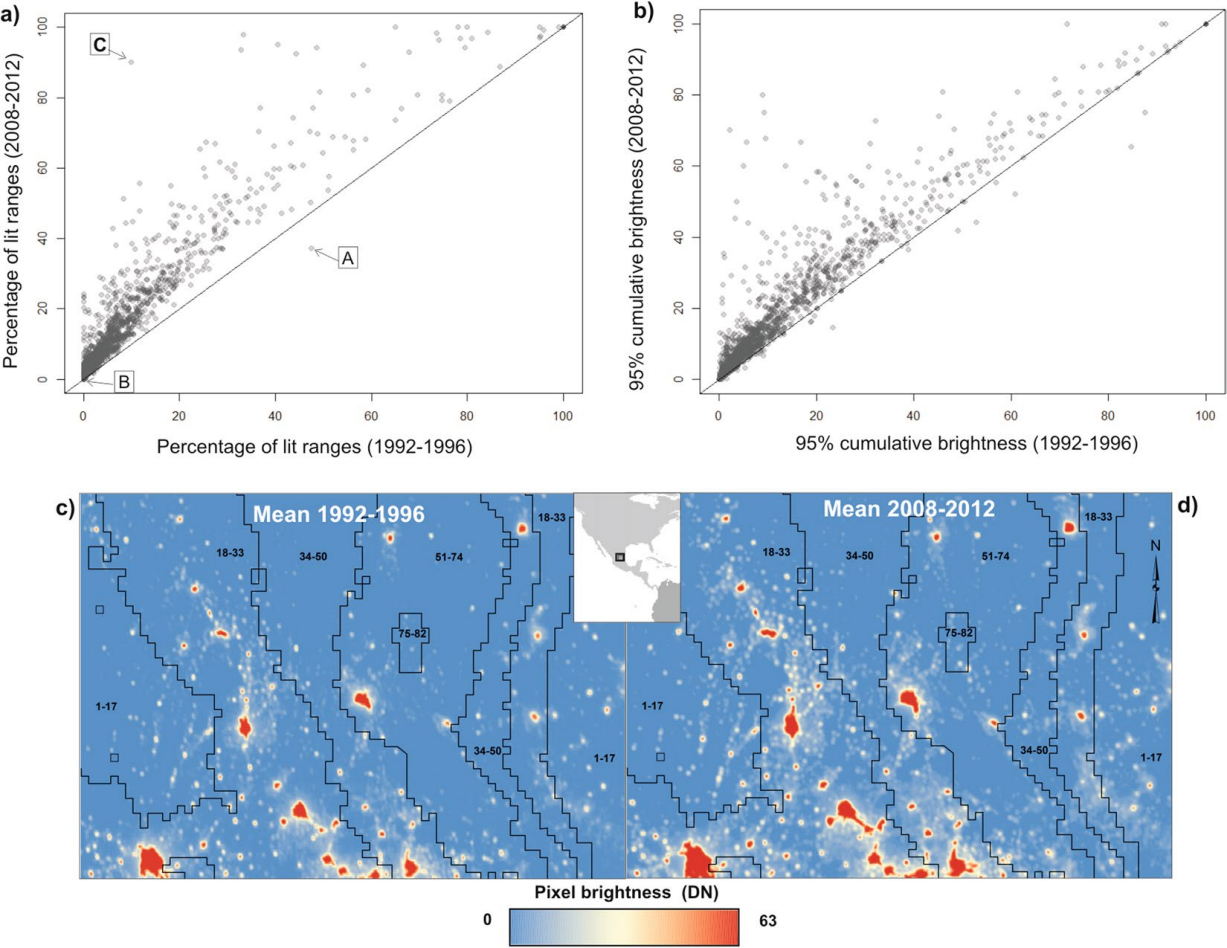
Changes in the artificial lighting of the ranges of cactus species. (**a**) The relationship between the percentage of the geographic range of each cactus species that was lit in 1992–1996 and in 2008–2012. (A) *Echinocactus grusonii*, (B) *Sclerocactus nyensis* and (**C**) *Leptocereus leonii*. (**b**) The relationship between the percentage of pixels contributing to 95% of the cumulative digital number (ΣDN) within the geographic range of each cactus species in 1992–1996 and 2008–2012. The solid line is that of equality in both figures. (**c**) and (**d**) mean nighttime light for the two periods analysed (1992–1996 and 2008–2012) respectively, on five classes of the cactus species richness. Areas with 1 to 17 species are noted by the class 1–17 and so on for the other classes.

For almost all cactus species the percentage of their geographic range that was lit increased from 1992–1996 to 2008–2012, often markedly (e.g. *Leptocereus leonii*, Fig. 3a). Indeed, this percentage only declined for 10 species (*Echinocactus grusonii, Echinocereus barthelowanus, Escobaria hesteri, Escobaria minima, Hylocereus extensus, Parodia buiningii, Parodia neohorstii, Sclerocactus nyensis*, and *Yavia cryptocarpa*). The decrease for *E. grusonii* could be explained by the fact that most of its range was converted into a dam in 1993, and therefore urban expansion and road development were stopped in the area. Also, the area where *Echinocereus barthelowanus* is found was subject to mining activity, which apparently stopped or decreased during 2008–2012. For the remaining species, the reasons are currently unexplained. For the majority of species (89%), a greater proportion of total pixels of the geographic range contributed to 95% of the cumulative DN when averaged over the last five years of the time series compared to the first five years (Fig. 3b). That is, ALAN is increasingly uniformly disseminated through their ranges.

**Figure 4:**
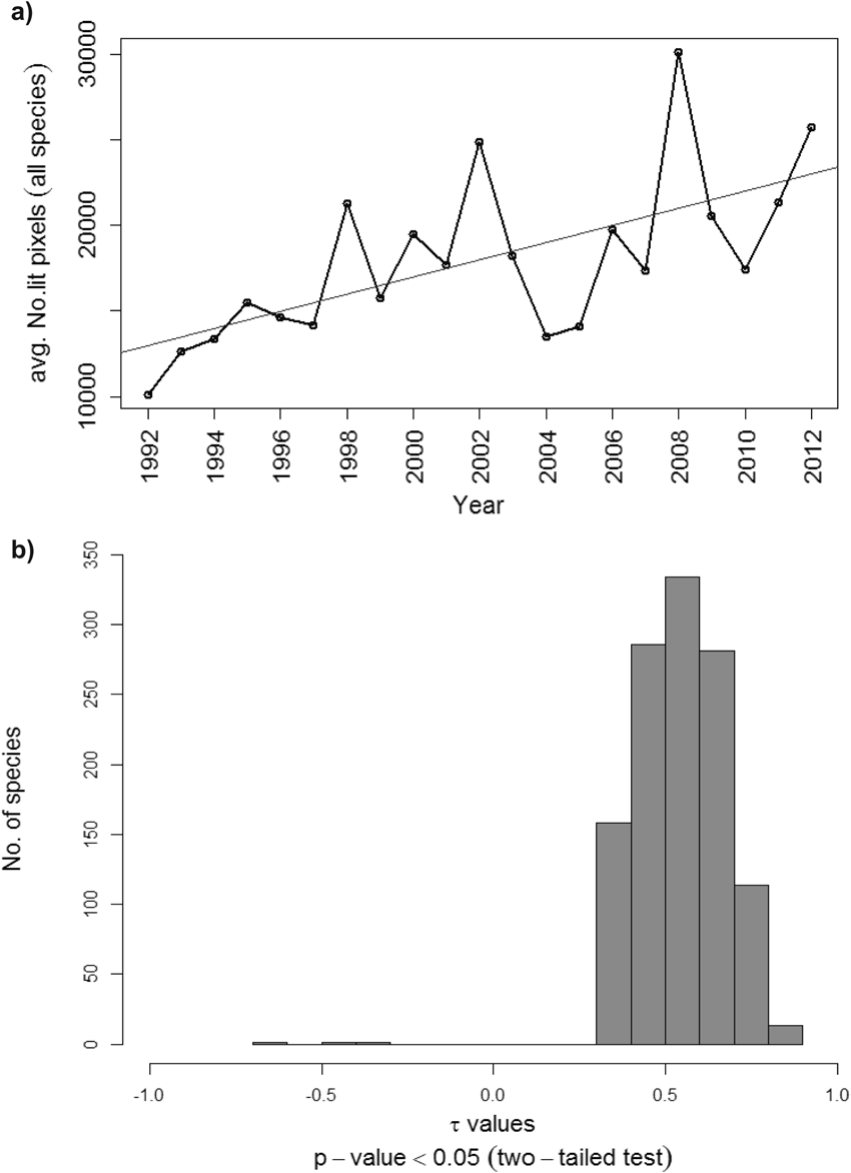
Trends in artificial lighting of the ranges of cactus species. (**a**) Temporal trend in the average number of lit pixels calculated across all cactus species during the 21 years; (**b**) number of cactus species with significant positive (1,186 species) and negative tau values after performing a Mann-Kendall Trend Test. Significant negative tau values correspond to *Echinocactus grusonii* (tau = −0.64), *Sclerocactus nyensis* (tau = −0.43) and *Escobaria minima* (tau = −0.32).

The average number of lit pixels for all cactus species during 1992–2012, calculated by considering the average number of lit pixels for all the species in each of the 21 years, showed a significant upward trend (tau = 0.52, p-value < 0.001; Fig. 4a). This was confirmed by testing each species individually. For 1,186 cacti, the Mann-Kendall trend test gave positive tau values ranging from 0.31 to 0.85 (p = 1.19 × 10^−7^ to <0.05, n = 1,435; Fig. 4b). A significant downward trend was found for three species: *Echinocactus grusonii* (tau = −0.64, p-value < 0.001), *Sclerocactus nyensis* (tau = −0.43, p-value = 0.007), and *Escobaria minima* (tau = −0.32, p-value = 0.04). For a further 246 species there was no statistically significant temporal trend. There was a significant negative relationship between the species richness of an area and its DN value for the first (1992–1996; rho = −0.21, 95% C.I: −0.24 to −0.17; p-value < 0.001) and for the last five years (2008–2012; rho = −0.19; 95% C.I: −0.22 to −0.16; p-value < 0.001) of DMSP-OLS data analysed (Supplementary Figure S1). A similar relationship was found for threatened species richness for both periods (1992–1996, rho = −0.16, 95% C.I: −0.24 to −0.1, p-value < 0.001; and for 2008–2012, rho = −0.2, 95% C.I: −0.21 to −0.06; p-value < 0.001, Supplementary Figure S1).

**Figure 5:**
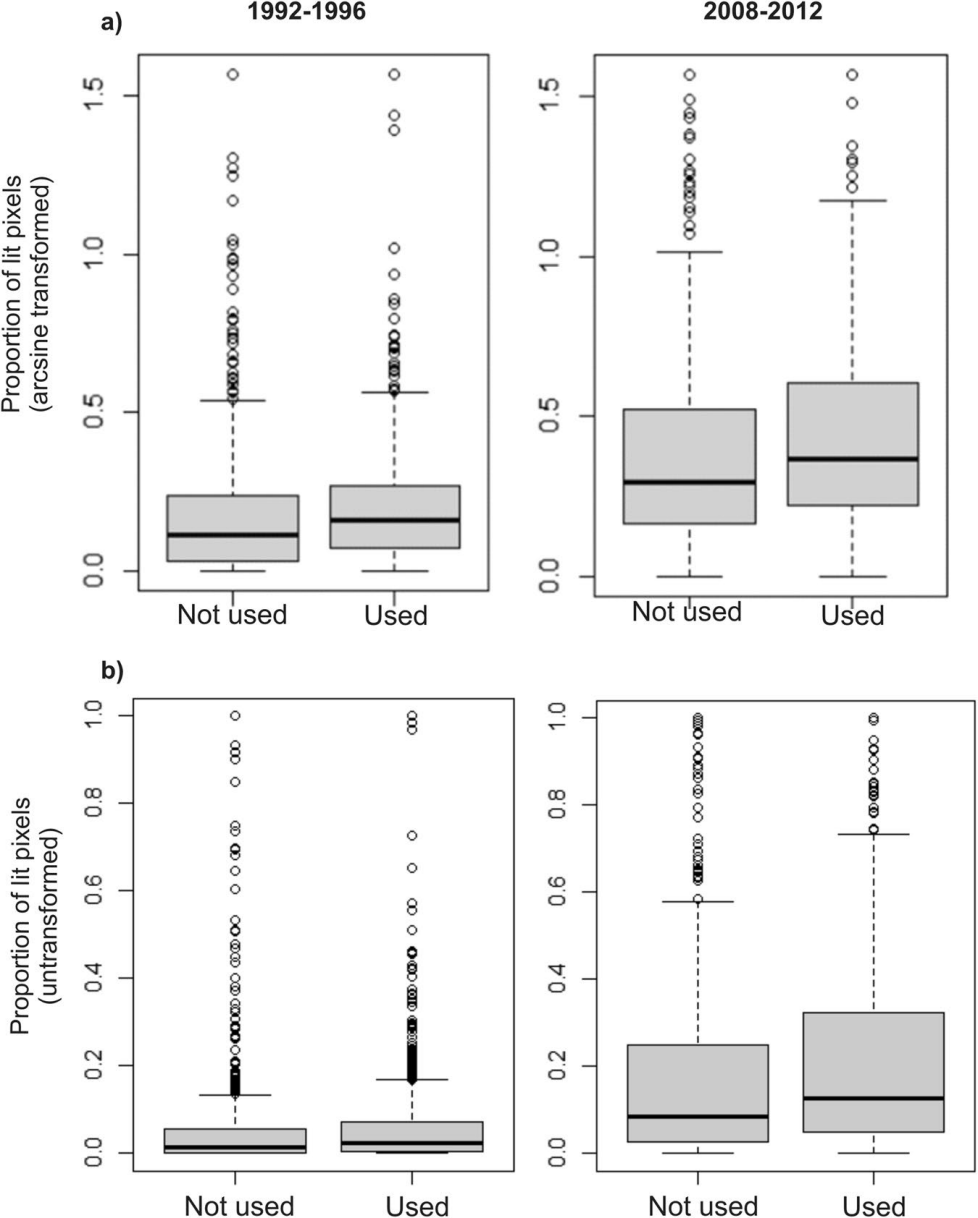
Artificial lighting and cacti use by people. Average proportion of lit pixels for (**a**) arcsine transformed and (**b**) untransformed data in the geographic ranges of cactus species that are (838 species) and are not used by people (597) during the two periods analysed.

During the period 1992–1996, cactus species that are used did not show a significantly higher proportion of lit pixels in their geographic ranges than did those that are not used (F_(1,1432)_ = 2.71, p-value = 0.1, 95% C.I.: −0.003 to 0.041; Fig. 5a). This contrasts with the period 2008–2012, for which cacti that are used had a significantly higher proportion of lit pixels in their geographic ranges (F_(1,1432)_ = 6.23, p-value = 0.01, 95% CI: 0.009 to 0.77; Fig. 5a).

## Discussion

Here we show for the first time the extent to which ALAN is co-occurring with the global distribution of a whole family of plant species: the Cactaceae. We further demonstrate how this co-occurrence has increased in recent decades, both in terms of the number of species whose geographic ranges are overlapped and also in terms of the proportion of those ranges that are becoming encroached by ALAN. Indeed, our results show that the vast majority of cacti (89.7%) had parts of their ranges already experiencing ALAN in 2012. This is a higher percentage than documented for mammals, which amongst vertebrates are of particular concern with regard to ALAN, given the predominance of nocturnal species in the group and the high level of extinction risk that many of them already face^23^. The observed increasing trend of ALAN in cacti ranges fits with the results reported from the Global Cactus Assessment^25^ which showed that two main sources of ALAN, that is residential and commercial development, and mining and quarrying, are the third and fourth most predominant threats affecting cacti. Residential and commercial developments are mainly disturbing species located in coastal areas such as the Baja California peninsula in Mexico, the Caribbean, and the southern coast of Brazil. Mining and quarrying affect several species in the cactus rich areas of eastern Brazil and northern Mexico. The spread of brightness within the ranges of cactus species might be an indication of the development of these activities during the time period covered by the analysis. These anthropogenic activities are also having a differential effect on the species that are used, for which distribution areas are experiencing a greater increase in exposure to ALAN than for species that are not used. However, more comprehensive studies are required to identify locally if species that are managed in wild and/or in anthropogenic created spaces are most affected.

Whilst based on the best long-term ALAN and cactus distribution data available, the analyses presented may actually underestimate the extent to which natural darkness has been eroded in the geographic ranges of cacti and the potential impacts. Although the species range data are quite coarse relative to the ALAN data, potentially inflating observed levels of overlap between the two, this effect is likely to be outweighed by the fact that a conservative detection threshold was used for ALAN (see Methods) and that the ALAN data do not capture the full extent of skyglow, which may propagate emissions very far from the source^33^. This may result in a substantial underestimate of the overlap. This said, ALAN values for some pixels, particularly those with measureable but low levels of nighttime lighting, may result from isolated lights rather than ‘pixel-wide’ illumination. This may exacerbate observed overlap.

ALAN could have both positive and negative effects on cacti. The light requirements for successful germination, growth, fertilization and dispersal of plant species in general vary greatly. This is no less true of cacti, providing opportunities for ALAN to have diverse impacts. Early research performed on cactus species revealed different responses of germination to ALAN. For *Carnegiea gigantea*, germination of seeds exposed to red and far-red light in a lighted room was higher than under dark conditions^34^. *Mammillaria longimamma*, and *Helianthocereus pasacana* germinated in the dark while *Parodia maassii* required long exposure to light^35^. More recent studies have sustained the importance of light and/or darkness for germination^36,37^ (but see^31^ for a review). The requirement for light to trigger the interruption of seed quiescence provides competitive advantages in diverse species and can be a determinant of the structure of plant communities^38–40^. Ben-Attia *et al*.^41^ have also shown that lunar phase and in particular full moon light might influence blooming in the cactus *Cereus peruvianus*, suggesting that ALAN could alter blooming patterns in cactus species.

Cacti have crassulacean acid metabolism (CAM) which enables plants to improve water efficiency by opening stomata at night and keeping them closed during the day, the hot and drier period. It is well known that the amount of light received by CAM plants has an effect on the opening of stomata^42^, affecting the balance between CO_2_ fixation and the accumulation of organic acids^43,44^. Exposure to ALAN by cactus species might induce an extension of the time for which stomata are closed, triggering less efficient CO_2_ fixation. CAMs contribution to total CO_2_ fixation depends on many factors, from differences in genotypic expression to variable environmental conditions^45,46^, and disentangling the effect that ALAN may have on this process is not an easy task. Although there is no direct published evidence that ALAN can have an effect on the CAM process, there is evidence of the differential effects of photoperiod on gas exchange and growth of cactus species. For instance, by increasing photoperiod from 6 to 18 h in *Ferocactus acanthodes* and *Opuntia ficus-indica* growth increased by 81% and 50% respectively^47^. Variation in daily incident photosynthetic photon flux resulted in differences in elongation of cultivated *Hylocereus undatus*^48^. Experiments on the cultivated ‘crimson giant’ (*Hatiora gaertneri)* showed that photoperiod and temperature are critical for controlling flowering^49^. ALAN might have an effect on CAM processes in wild cactus species altering growth rate and flowering timing, which may alter individual fitness, changing population dynamics and ultimately modifying community composition.

ALAN is likely also to have indirect effects on cacti, including via impacts on pollinators and dispersers. Pollinators of cacti include insects, birds and bats^32^, all groups whose behavior has been shown to be vulnerable to influences by ALAN^11,50–52^. Empirical observations of the lesser long-nosed bat (*Leptonycteris curasoae*), a species that pollinates columnar cacti, have shown that it prefers environments with lower light intensities for foraging movements^53^. In addition, studies^32,54,55^ report that the majority of species of columnar cacti (Tribe Pachycereeae) found in Mexico (70 species) are bat pollinated (72%). Alterations to the behavior of *Leptonicteris curasoe* due to ALAN, e.g. by avoiding individuals of cactus species in lit areas, might cause changes in cacti at individual and/or at community level by modifying pollination and dispersal rates. Also two main groups of dispersers of cacti have been recognized^56^. Primary dispersers take fruits directly from the plant during the day (e.g. birds, lizards) and night (e.g. bats). Secondary dispersers take the fruits from the ground (e.g. ants and rodents), and again bats and rodents are likely often to be nocturnal; rodent behavior has been shown often to be strongly shaped by ALAN^8^.

Although the negative relationships documented between the species richness of cacti (and of threatened cacti) and levels of ALAN are suggestive that high levels of ALAN in an area may not be conducive to a high biodiversity of cacti, it remains challenging to discriminate the particular influences of ALAN on patterns of species richness from those of other factors that are commonly associated with the introduction of ALAN into the environment, especially broader habitat change. Nevertheless, increasing evidence of the effects of ALAN on a diversity of taxa^8^ and on communities and ecosystems^57^ call attention to the necessity of disentangling its effects.

Cacti are perceived as amongst the most charismatic of plant taxa, emblematic of arid lands, and of major cultural significance^25^. They may thus be of particular concern in terms of the impacts of ALAN, especially given the high proportion of species experiencing the erosion of natural darkness within their geographic ranges. However, there is little reason to believe that such changes are atypical of those that are being experienced by many other groups of organisms.

## Methods

### Data

ALAN data were derived from the global nighttime light composite images from the Defense Meteorological Satellite Program’s Operational Linescan System (DMSP-OLS), which currently provides the only available global scale long time series data suitable for analysis of the changing trends in ALAN. These images (available from www.ngdc.noaa.gov/eog/download.html) are annual cloud-free composites of detectable stable light sources on Earth. They are produced at ~1 km resolution (resampled from data at 2.7 km resolution) for the years 1992–2012. Each pixel value is represented by a digital number (DN) of between zero and 63. A value of zero represents darkness, while very brightly lit urban areas typically saturate at 63. The DMSP-OLS composites are not radiometrically calibrated, and geolocation errors exist in the final products, leading to geographic inconsistencies. In addition, original data were acquired from six different satellites with different sensors, so these images should be cross-calibrated before performing any multi-temporal analysis. An empirical procedure developed by Elvidge *et al*.^58^ considers “stable” areas (no apparent lighting change areas) to calibrate across years by developing a second-order regression function. However, finding areas with no lighting change over time and with a full range of DN values at continental scales may lead to important inaccuracies given the variation across areas at this scale. In addition, the coefficients of ordinary least-squared regressions are markedly influenced by outlying values. Li *et al*.^59^ used a robust regression technique which iteratively removes outliers avoiding this problem. Here we followed Bennie *et al*.^60^ who used a quantile regression through the median, a form of robust regression which is insensitive to outlier values.

Data on almost all known cactus species were obtained from the Global Cactus Assessment (GCA^25^). During this exercise, following a standardized IUCN methodology, the world’s leading experts compiled data for each extant species on its distribution, population trends, habitat, ecology and threats, and evaluated its conservation status. All data collated during the assessment process are publicly available on the IUCN Red List website (http://www.iucnredlist.org/). The present work is based on the 1,435 cactus species for which range maps are available (of a total of 1,480). Following the GCA, we distinguished between those cacti that are utilized by people and those that are not, particularly as it seems likely that the former are differentially exposed to ALAN (assuming a closer proximity on average to sources of artificial light). We included 10 broad categories of use: construction, animal food, human food, fuel, handicrafts, medicine (human and veterinary), other household goods, horticulture, specimen collection and other.

### Data Processing

All data were re-projected to the Behrmann equal-area projection to perform analyses. Averaged intercalibrated DMSP images were calculated for the first five years (1992–1996) and for the last five years (2008–2012) of the time series using the DN values. We then extracted the DN values within the geographic range of each cactus species for the whole time series and for the two periods separately. The average intercalibrated DN value was also calculated for each species for all years. The last process is preferred over the use of a single year’s value to soften any problems of error variation in the number of lit pixels through time.

Following Gaston *et al*.^61^, we defined a threshold for ‘darkness’ of less than 5.5 DN. This threshold was established after the finding by Bennie *et al*.^60^ that 94% of observed increases in DN of more than 3DN and over 93% of observed decreases of the same magnitude could be attributed to a known change on the ground consistent with the direction of change (i.e., urban expansion, industrial closure). The threshold of <5.5DN (or <6DN as a round value) is effectively twice the detection limit for change in DN, and thus provides a conservative estimate of the extent of ALAN due to noise in the data set or calculation errors^61^. The extent of ALAN found within the geographic range of individual species was assessed by calculating the proportion of lit pixels (the number of lit pixels/total number of pixels). The 95% cumulative brightness, measured as the cumulative DN or ∑DN in the range of each cactus species was calculated to determine the dissemination of ALAN within. In addition, two integrated cacti distribution maps were created: 1) a species richness map and 2) a threatened species richness map. The species richness map was created by overlapping all current individual cactus species distribution areas. The same procedure was used to make the threatened species map. Then, for both, each richness area was in turn subdivided and used as a mask on the averaged DMSP image for the last five years (2008–2012). Each richness area was defined as the number of species allocated in one pixel (pixel area = 65.75 ha), resulting in 82 richness areas, so that the lowest richness area, contains one species and the highest richness area contains 82 species. We obtained 82 different images, and we show the distribution of species richness for the whole family and for the threatened cacti species in two separate maps (Fig. 1a,b). We analysed 417 threatened species, each categorized under one of the IUCN threatened categories: Critically Endangered, Endangered or Vulnerable (IUCN, 2001). For the threatened species richness map we obtained 14 different images, which correspond to richness areas ranking from 1 to 14. Pearson’s product moment correlation was determined for the relationship between species richness and the averaged DN values from 2008–2012. Threatened species richness was tested in the same way.

We tested whether an upward or downward trend in ALAN was occurring in the geographic ranges of each of the 1,435 cactus species by considering the mean of the number of lit pixels for each year for the entire time period (21 years of the DMSP-OLS composites) and applying a Mann-Kendall trend analysis. This is a test for monotonic trend in a time series based on the Kendall rank correlation and tau^62^. The evenness of the nighttime light composite within the geographic range of each species was measured by examining the proportion of total pixels that contributed to 95% of the cumulative DN found within them (∑ DNs).

To evaluate whether there was a significant difference in the amount of ALAN in the geographic ranges of cacti that are used by people compared with those that are not, we applied a general linear model with the proportion of lit pixels (calculated as the number of lit pixels averaged for both periods, 1992–1996 and 2008–2012, and then divided by the total range size per each species) as the dependent variable and a single fixed factor describing if the species was used or not. The proportion of lit pixels was arcsine square root transformed before analysis to meet with linear regression assumptions. All data processing was performed using the statistical package R^63^. Raster images were analysed using the packages ‘raster’^64^ and ‘rgdal’^65^. The scatterplot with heat-density colours was created using the ‘LSD’^66^, ‘MASS’^67^, and ‘colorRamps’ packages^68^. The trend test was performed using the ‘MannKendall’ function implemented in the Kendall package^62^.

## Electronic supplementary material

Supplementary information
