# Supplementary material for: Erosion of natural darkness in the geographic ranges of cacti
Source: Sci Rep. 2018 Mar 12;8:4347. doi: 10.1038/s41598-018-22725-8 (PMC5847551; doi:10.1038/s41598-018-22725-8)
Supplement: Supplementary file 1 — Supplementary information [file 41598_2018_22725_MOESM1_ESM.pdf]

Erosion of natural darkness in the geographic ranges of cacti

Maria Eugenia Correa-Cano[1*], Bárbara Goettsch[2], James P. Duffy[1], Jonathan Bennie[1], Richard Inger[1] and Kevin J. Gaston[1]

[1]Environment and Sustainability Institute, University of Exeter, Penryn, Cornwall TR10 9FE, UK

[2]International Union for Conservation of Nature, Global Species Programme, The David Attenborough Building, Pembroke Street, Cambridge CB2 3QZ, UK

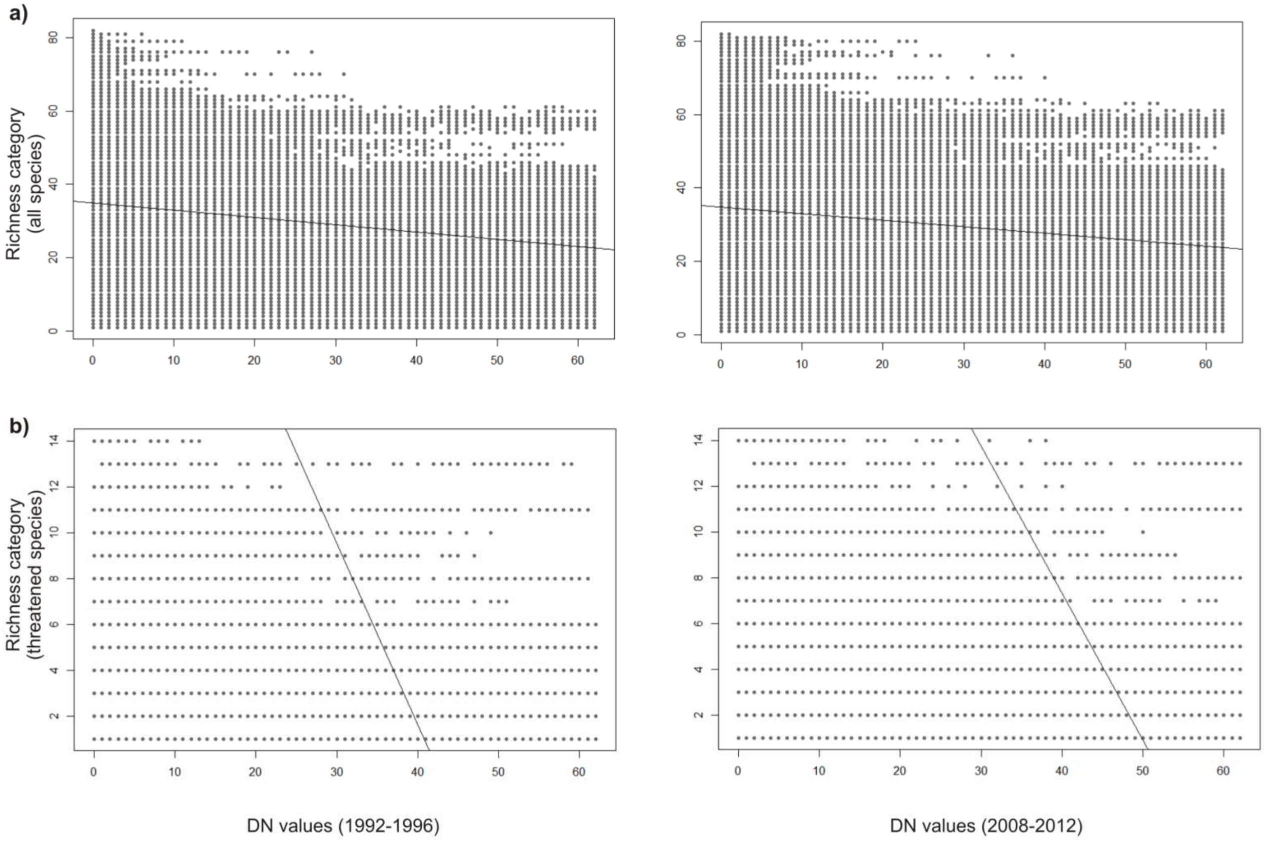

Supplementary Figure S1 Artificial lighting and species richness. (a) Relationships between species richness categories and its average DN values for the periods 1992-1996 and 2008-2012 and (b) relationships between threatened species richness categories and its average DN values for the same periods of time.
